# Light, More Light

**Published:** 1893-07-29

**Authors:** 


### A Physiological Lantern.

### I.—"LIGHT, MORE LIGHT."

Perhaps it would be impossible to find a man anywhere who could contend that we have too much light on man, his origin, his place in nature, his duty, and his destiny. There are two ways of yielding obedience to the ancient oracle, "Know thyself"—the internal way and the external. For more than five thousand years the world has used chiefly one way, the internal. That is the method which religion employs now, and has always employed. Philosophy, also, at any rate in past times, has always "enquired within." To-day she looks wistfully in the direction of the external method, and begins to ask herself, not without trepidation, whether a combination of the two might not after all be the real organon of truth.

The question of method is really a question of aim. What does a man want to be at? Is logic-chopping his chief business, or does he desire to find a foundation of truth upon which he may build something of worth and permanence? For the mere logic-chopper we have no manner of regard, how clever soever he may be. At best he is a parrot, and that of the driest and most exasperating order. Now it is possible to conceive a parrot so clever, and so incessant in his endeavours to talk all day, that even his admiring owner might at last come to think that the fittest thing to be done with him would be to "wring his neck." Certainly some of the "philosophers" of the past qualified themselves for that distinction, and it is not impossible that some of the present and of the future may prove themselves worthy of the same ignominious fate.

Physiology tenders her services as a promoter of sanity. She promptly deposes logic from his sovereignty, and puts truth, objective, tangible, discoverable, provable truth in his place. "Out upon you," she says to Logic! "What have you been doing these five thousand years, you chattering daw? You have been doing nothing, and that which is worse than nothing! You have been leading the world through all manner of bogs and quagmires, and have left it stranded in a region of hopeless darkness, from which it is doubtful whether you or anybody else will ever be able to get it out!"

But physiology is a "new broom," and "new brooms" proverbially "sweep clean," too clean, perhaps, sometimes; they are apt to brush away the nap of the carpet, even the carpet of truth. Physiology has been more than a common broom; she has been a scouring, street-sweeping, pavement-wearing machine in many hands. She has sometimes torn up macadam by the roots, and made good roadways impassable. We must not, therefore, have too much of physiology, or, at least, we must teach her, as we teach Mary Ann, "to know her place."

Our aim is practical; we confess it. Truth for truth's sake is good; truth for man's sake is better. Right physiology, like right religion, has a message for the world. Right physiology, like right religion will never be content until every man is her disciple. A physiology of the cloisters is a physiology of the night, of the witch's cell, of the mystery-monger's arcana diabolica. In a physical, and to a considerable extent, in a mental and a moral sense, physiology is "the light of the world."

The boast of physiology is that she uses her eyes, and it is a very legitimate boast, within limits, for it is not, after all, the eye that she works with, but the brain. It is not the eye that sees, that perceives, it is the brain. The eye is merely the instrument which carries the impression to the brain, as the spoon is the instrument that carries the food to the mouth. It is the mouth that eats, not the spoon. So, even in things physiological, it is the brain that sees and perceives, not the eye. But this is bringing us awkwardly near the "internal" method of the old-fashioned philosopher, is it not? It is; but it is the philosopher's method with a difference.

Are there, then, two methods by which men arrive at truth, or only one? There is only one, but it consists of two parts. We look and we think, that is our method. The man who looks but does not think masters no truth, and the man who thinks but does not look masters none—or, at any rate, very little. Second-rate physiology looks much but thinks little. First-rate physiology thinks as much as it looks, and often a great deal more. In short, the true physiologist is a philosopher as well as a student of physiology. He who is no philosopher is no physiologist.

Physiology is a lantern, a light which helps us to see things we could not see without it. Now there are lanterns and lanterns. The old-fashioned stable-lantern was glazed with a dim kind of talc or horn. You could see a big creature like a horse with its aid if you looked carefully, but it made no revelation of a black-beetle, hardly even of a skulking rat. The policeman's bull's-eye is a lantern of a very different order. It brings everything into full vision; the glare of the burglar's eye and the glitter of the steel on his furtive weapon. Physiology is a policeman's bull's-eye to those who know how to use it. We must all admit that the world is a pretty confusing place in these late times. What with the practical difficulties of the present, and the accumulated confusions of the past, it is no easy thing for a man to pick his way smoothly and safely through it. A good bull's-eye is a useful friend in such dangerous darkness.

Physiology devotes her best energies to the study and elucidation of man. Herein she is like philosophy and like religion. Philosophy says "the proper study of mankind is man." Religion affirms that "the very hairs of man's head are all numbered" by the highest intelligence. It is true that physiology stands aside periodically from her main task, and studies organic life in all its forms. But when everything is said, the chief riddle she sets herself to answer is the riddle of man. "What is truth?" said Pilate. "What is man?" says Physiology. How came he into being? Through what stages has he passed? What is his present business in the world? What is his worth? By what standards shall his worth be judged? What are his future prospects? Is he a candle which will burn down and die out, or which may be prematurely snuffed out in the process? Or is he a gleam of intellectual light glowing brightly or dimly in a material lantern which will continue to grow when the material lantern is reduced to its constituent elements? Above all, is he the subject of law, and of what law? Are there any duties he should imperatively perform? Are there any offences he should as imperatively avoid? What are the duties to be done? What are the offences to be avoided? What are the penalties to be dreaded? What are the rewards to be won? Physiology is the newest of all the lanterns which the ages have turned upon man; and religion is the oldest; and philosophy stands midway between the two. What religion has said of man we know; and we sometimes think we know what philosophy has said. Let us try to hear what it is that physiology has now to say. We purpose during the next six months to publish an occasional article with the object of showing how important is the bearing which physiology has upon some of the most venerable problems in religion and philosophy, as well as upon many of the most urgent practical questions in the common and special morals of our own time. That the body has much to do with the mind is commonly believed. We propose to show in fuller detail how intimate and all-pervading this relation between organism and character really is.

